# Development of Electrochemical DNA Biosensor for Equine Hindgut Acidosis Detection

**DOI:** 10.3390/s21072319

**Published:** 2021-03-26

**Authors:** Joshua Davies, Carol Thomas, Mohammad Rizwan, Christopher Gwenin

**Affiliations:** 1 School of Natural Sciences, Bangor University, Bangor, Gwynedd, Wales LL57 2UW, UK; jjdavies1995@hotmail.co.uk (J.D.); carol.thomas@bangor.ac.uk (C.T.); m.rizwan@bangor.ac.uk (M.R.); 2 Department of Chemistry, Xi’an Jiaotong-Liverpool University, 111 Ren’ai Road, Suzhou Industrial Park, Suzhou 215123, China

**Keywords:** *Streptococcus equinus*, *Mitsuokella jalaludinii*, laminitis, DNA hybridization, equine hindgut acidosis, electrochemical biosensor, veterinary diagnostics, point-of-care testing

## Abstract

The pH drop in the hindgut of the horse is caused by lactic acid-producing bacteria which are abundant when a horse’s feeding regime is excessively carbohydrate rich. This drop in pH below six causes hindgut acidosis and may lead to laminitis. Lactic acid-producing bacteria *Streptococcus equinus* and *Mitsuokella jalaludinii* have been found to produce high amounts of L-lactate and D-lactate, respectively. Early detection of increased levels of these bacteria could allow the horse owner to tailor the horse’s diet to avoid hindgut acidosis and subsequent laminitis. Therefore, 16s ribosomal ribonucleic acid (rRNA) sequences were identified and modified to obtain target single stranded deoxyribonucleic acid (DNA) from these bacteria. Complementary single stranded DNAs were designed from the modified target sequences to form capture probes. Binding between capture probe and target single stranded deoxyribonucleic acid (ssDNA) in solution has been studied by gel electrophoresis. Among pairs of different capture probes and target single stranded DNA, hybridization of *Streptococcus equinus* capture probe 1 (SECP1) and *Streptococcus equinus* target 1 (SET1) was portrayed as gel electrophoresis. Adsorptive stripping voltammetry was utilized to study the binding of thiol modified SECP1 over gold on glass substrates and these studies showed a consistent binding signal of thiol modified SECP1 and their hybridization with SET1 over the gold working electrode. Cyclic voltammetry and electrochemical impedance spectroscopy were employed to examine the binding of thiol modified SECP1 on the gold working electrode and hybridization of thiol modified SECP1 with the target single stranded DNA. Both demonstrated the gold working electrode surface was modified with a capture probe layer and hybridization of the thiol bound ssDNA probe with target DNA was indicated. Therefore, the proposed electrochemical biosensor has the potential to be used for the detection of the non-synthetic bacterial DNA target responsible for equine hindgut acidosis.

## 1. Introduction

An excess of carbohydrates in the diet can lead to the onset of laminitis in horses, a condition where the distal phalanx and the inner hoof wall fail to attach, resulting in damage to the hoof and the surrounding area [1]. Failed attachment causes the distal phalanx to be forced down into the sole of the hoof under the horse’s weight [2]. This leads to lameness, and often euthanasia of the horse as a result [1,2]. Laminitis occurs in roughly 7–14% of horses worldwide and is usually diagnosed from the observation of certain behavioral patterns such as an unwillingness to walk, uneven distribution of weight, and lameness [3]. By this point, the health of the horse may have deteriorated to the point of permanent anatomical damage, highlighting the need for earlier detection [4]. Racehorses, in particular, suffer from metabolic issues because of their dietary setup which consists of short bursts of concentrate feeding in between lengthier periods of grazing [5]. This carbohydrate-intensive feeding regime can cause the bacterial population in the hindgut to change as quickly as five hours post concentrate feed leading to equine hindgut acidosis and other similar metabolic problems [5]. When the pH of the hindgut is lowered by the formation of volatile fatty acids, the bacterial population is altered [1]. The population skews in the favor of lactic acid producing bacteria, lactic acid accumulates, and fiber fermenting bacteria are suppressed [6]. As the pH drops below six, the horse can be diagnosed with clinical hindgut acidosis [5].

The virginiamycin treatment used for controlling equine hindgut acidosis and laminitis has limitations, and so early detection of lactic acid producing bacteria could reduce the need for this treatment [7]. The abundant increase of two bacteria, namely *Streptococcus equinus* (SE) and *Mitsuokella jalaludinii* (MJ), post concentrate feed makes them suitable targets for detection and subsequent indicators of equine hindgut acidosis. However, the method of bacterial detection must be highly specific to the bacterial groups and it must be a rapid and portable technique that can be utilized at the horse’s locations. These requirements make biosensors suitable for detection of the target bacteria. In addition, the principle of the nucleic acid type biosensors is the exploitation of the interaction of complementary base pairs [8,9]. Utilization of nucleic acids for biosensing applications initially generated interest due to their high specificity [10,11]. This high specificity along with the lack of lengthy purification processes and comparatively low costs makes nucleic acids ideal candidates for this work. Historically, deoxyribonucleic acid (DNA) hybridization has been monitored by methods such as gel electrophoresis, membrane plots, etc.; however, these methods are too laborious to achieve such goals efficiently [12]. Biosensors have gathered interest because of their ability to quickly detect events such as DNA hybridization and the fact that they are cheaper and faster alternatives to more traditional methods [12]. Therefore, the capture probes in this work were designed to be complementary to the two specific bacterial genomes of interest.

The most commonly used transduction element for nucleic acid biosensors is optical due to the relative ease of tagging nucleic acids with a fluorescent tag [13,14]. However, the need for labelling can be time consuming, and for applications that require quick and mobile detection, these methods are not suitable [15]. For these applications, label-free electrochemical methods are more suitable and have been utilized due to their portability and high sensitivity to changes at the surface of the electrode [13,16,17,18]. Additionally, the diversity of available electrodes, paired with the fact that no expensive transduction equipment is required due to the direct signal produced, means the costs for electrochemical methods can be relatively low [10,13,19,20,21]. Electrochemical DNA hybridization biosensors generally work by monitoring the current response of a DNA hybridization event where an electrode modified with a single stranded DNA (ssDNA) capture probe layer is introduced into a solution of its complementary target ssDNA [13]. The formation of double stranded DNA (dsDNA) through hybridization is often detected through a change in the current of a redox probe [x]. Because of the need for portability and rapid detection of the chosen bacteria, label-free electrochemical methods are most suited to this work, and therefore, electrochemical impedance spectroscopy (EIS) was chosen as the basis of this DNA hybridization biosensor.

EIS is a technique that can rapidly detect events such as DNA hybridization, and changes in the shape and structure of DNA, etc., at the surface of the working electrode [22,23]. EIS utilizes a redox probe at the surface of the working electrode and measures the probe’s ability to undergo a redox reaction [16]. The changes in impedance at the working electrode, when modified with ssDNA and when hybridization to the target ssDNA occurs, could be monitored to confirm successful detection of the DNA target in a sample [24]. As impedance-based DNA sensors do not require redox labels [16,22]. Further, the technique benefits from its simplicity, lower expense, and ability to be used on smaller portable electrochemical units [22].

Electrochemical biosensors often rely on the formation of self-assembled monolayers (SAMs) [25,26]. In particular, SAMs of thiol-modified compounds have been used extensively because the affinity between gold and Sulphur can be exploited [27,28]. Thiol containing compounds in contact with a gold electrode surface results in the spontaneous formation of a SAM of thiol bonds bound across the surface [29]. It is accepted that the binding interaction is a gold–thiolate bond and when binding with a reconstructed, gold substrates–Au(111) surface, the Sulphur group is thought to occupy a hollow site between three gold atoms in a hexagonal close packed (hcp) arrangement [27]. The interaction of an electrode bound DNA SAM with a DNA analyte has a considerable number of applications in fields including medical, pharmaceutical, and forensics, etc. [30]. These DNA bound surfaces have also had an application in the separation of chiral compounds such as proteins [29]. In electrochemical DNA hybridization biosensors, the oligonucleotide capture probe is often linked to the gold electrode surface with the use of a thiol group or an amine group because a SAM can be generated spontaneously [22]. The most widely studied example of this is the SAM of alkanethiols on a gold surface which can be modified to contain linkers that can conjugate with biomolecules [26]. The affinity for a SAM of Sulphur on gold, along with the relative chemical inertness of gold, means it is a very suitable electrode material for an electrochemical impedance-based DNA hybridization biosensor for the applications of this work [31].

However, currently available veterinary diagnostics are highly expensive, time consuming, require bulky and sophisticated instruments, and require a highly trained individual for their operation. For example, Loy et al. developed a multiplex real-time PCR assay for the detection of bacterial pathogens related to respiratory diseases in cattle using two thermocycling platforms [32]; whilst Rosa el al. used host biomarker-based ELISA to improve the diagnostic sensitivity of Mycobacterium avium subsp. paratuberculosis infection in cattle [33]. Furthermore, recently Gwenin et al. fabricated (a) a label free colorimetric assay for active botulinum neurotoxin using soluble N-ethylmaleimide-sensitive attachment protein receptor (SNARE) proteins (SNAP-25) conjugated gold nanoparticles [34], (b) a biosensor over gold working electrode for the detection of active botulinum neurotoxin in a real pharmaceutical sample using EIS [16], (c) SNAP-25 and vesicle associated membrane protein (VAMP) based EIS biosensors over a gold working electrode for detecting the activity of five botulinum neurotoxin serotypes (A–E) [17], and (d) a lateral flow device for the detection of sepsis pathogens using recombinase polymerase amplification [35]. In addition, Liu et al. used a nanocomposite of Au nanoparticles/toluidine-graphene oxide modified glassy carbon electrode for the fabrication of a label free electrochemical DNA biosensor to detect a multidrug resistance gene [36]. Moreover, to the best of our knowledge, there is no literature available for the electrochemical impedance-based DNA hybridization biosensor over gold electrode using a SAM strategy for equine hindgut acidosis detection.

**Figure 1:**
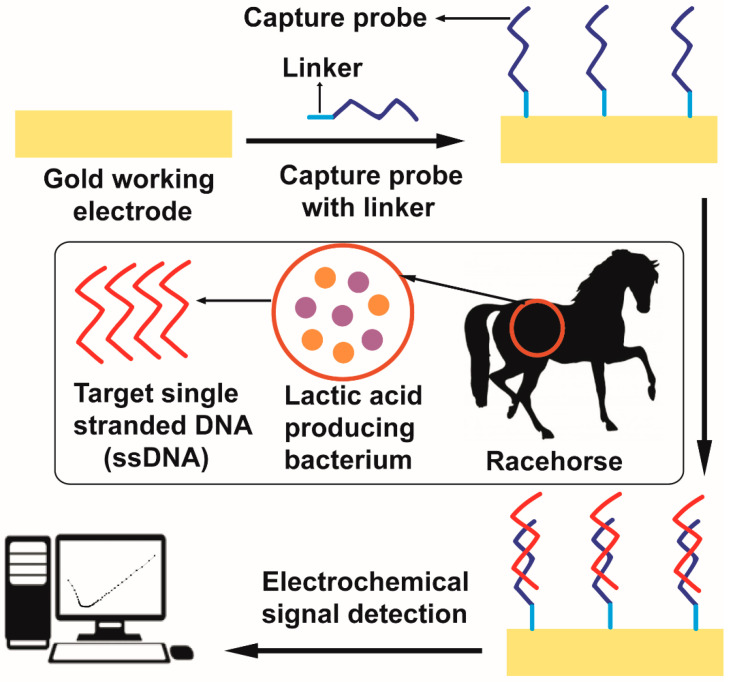
Electrochemical DNA hybridization biosensor fabrication.

Therefore, this work aims to utilize DNA hybridization as a way of detecting target ssDNA sequences found in the *Streptococcus equinus* and *Mitsuokella jalaludinii* genome by hybridizing that ssDNA with complementary designed capture probes. Henceforth, four types of different targets and capture probes combinations were designed and studied using polyacrylamide gel electrophoresis. Polyacrylamide gel electrophoresis study portrayed that only one capture probe (SECP1) and target (SET1) combination, among four different capture probe and target combinations, illustrated successful hybridization. Moreover, an adsorptive stripping voltammetry (AdSV) study demonstrated that thiol modified SECP1 bound more firmly over a gold electrode compared to unmodified SECP1. Therefore, an electrochemical biosensor was fabricated for the point-of-care testing of hindgut acidosis in horses. The process of electrochemical DNA biosensor fabrication for equine hindgut acidosis detection and monitoring is illustrated in Figure 1. Fabrication of electrochemical DNA hybridization biosensor was studied using both CV and EIS. Furthermore, the impedance generated by the hybridization event, in comparison to the impedance from the capture probe on the surface alone, will be used to prove that the sample contains the target bacteria.

These lactic acid producing bacteria are known to increase in population substantially in a matter of hours after a carbohydrate feed, making them valuable targets to detect. The proposed electrochemical biosensor could be used as a veterinary diagnostic for early screening for equine hindgut acidosis using natural DNA in real samples of horses to prevent cases and subsequent laminitis.

## 2. Materials and Methods

Gold working electrodes, gold coated glass subtrates, Au(111) were purchased from Arrandee Metal GmbH in Bremen, Germany. The synthetic target and capture probe oligonucleotides were purchased from Eurofins Scientific, London, UK. Additionally, the capture probe and target sequences were made up from lyophilized powder in 1× TE buffer (pH 8.0) for long term storage at −20 °C. −20 °C was selected for the DNA storage, as this temperature is sufficient for everyday use of DNA and DNA remains stable for six months to over a year. However, DNA would not be stable at higher temperature for more than a week, for example, at 4 °C. Moreover, to store DNA for years up to a decade, a temperature lower than −20 °C is required. For example, −80 °C could be used for years to a decade storage of DNA. All other chemicals and reagents were purchased from Sigma Aldrich, London, UK, or Thermo Fisher Scientific, London, UK, unless stated otherwise. The chemicals and reagents used throughout this work were of standard bio reagent grade or higher. All electrochemical measurements were performed using a Metrohm autolab potentiostat, PGSTAT 30 (Eco Chemie, Utrecht, The Netherlands) along with the Autolab Nova 2.1 software for measurement control.

### 2.1. Target Identification and Capture Probe Design

Initially, two types of bacteria were the focus of detection: *Streptococcus equinus* (SE) and *Mitsuokella jalaludinii* (MJ). To obtain target sequences for subsequent capture probe design, sequences from ten SE strains and six MJ strains were taken from the National Center for Biotechnology Information (NCBI). After identification of the target sequences, they were altered to contain up to four mismatched nucleotides. The ClustalW multiple sequence alignment tool was then used to check if the newly modified sequences aligned with the sequences of any other bacteria.

### 2.2. Polyacrylamide Gel Electrophoresis for DNA Hybridisation Study

Initially, hybridization of the capture probe and its corresponding target sequence, in solution, was monitored using in-house made 15% polyacrylamide gel electrophoresis. Bis-acrylamide (3 mL, 40 %), dH_2_O (3.344 mL), 1× tris-borate-ethylenediamine tetraacetic acid (TBE) buffer (1.6 mL), tetramethylethylenediamine (TEMED) (3 µL), and ammonium persulfate (APS) (56 µL, 10 %) were all added into a falcon tube and mixed with a pipette. Subsequently, the solution was added to the glass gel mound and a comb was inserted. The solution was then left for approximately 1 h until the gel had set. Once set, the gel was moved into a tank filled with 1× TBE buffer ready for samples to be loaded.

The ssDNA capture probe (5 µL, 10 µM) and target sequence (5 µL, 10 µM) were added to a micro-Eppendorf tube and pipetted up and down gently to homogenize the DNA solution. The solution was then allowed to react for 2 h at room temperature before the addition of 6× orange DNA loading dye (2 µL). Once the comb was removed, the hybridization solution (6 µL) was loaded into the wells of the set polyacrylamide gel. Additionally, capture probe (10 µM) and target DNA (10 µM) controls were loaded into the gel alongside the hybridization reaction (5 µL, 10 µM), 1× TE buffer (5 µL, pH 7), and 6× orange DNA loading dye (2 µL) were added to a separate micro-Eppendorf tube and mixed by pipette to form a capture probe control. 6 µL of both of these controls, along with a GeneRuler ultra-low range DNA ladder (5 µL), were added to separate wells in the gel. Once the samples were loaded, the gel was run at 100 V for 70 min to allow for sufficient separation and movement of the DNA. After the gel had been run, it was transferred into a weighing boat where it was stained with ethidium bromide doped TBE (15 mL, 1.25 µM) for 10 min. Once the staining process was complete, the ethidium bromide was drained off and the gel was visualized on the ultrasonic vibration potential (UVP) benchtop ultraviolet (UV) transilluminator, M-26V (Fisher Scientific, UK) for analysis. Alternatively, some of the polyacrylamide gels were stained with acridine orange (20 mL, 4 µM) for 10 min, then de-stained with distilled water for 15 min.

### 2.3. Pre-Treatment of ssDNA Capture Probes

To attach the thiol-modified ssDNA capture probe to the gold surface, any potential disulfide bonds that might have been formed in the capture probe solution during shipping had to be reduced into the thiol form. This was achieved by the use of the tris(2-carboxyethyl)phosphine hydrochloride (TCEP-HCl) reducing agent. To a micro-Eppendorf tube containing the lyophilized capture probe oligonucleotide, TCEP-HCl (200 µL, 10 mM) was added. This solution was shaken at room temperature (20–25 °C) for 60 min. Ammonium acetate (150 µL, 7.5 M) was added to the solution, followed by absolute ethanol to fill the Eppendorf tube. The solution was then shaken for 20 min at −20 °C followed by centrifugation for 5 min at 11,357× *g* to pellet the DNA. The supernatant was removed, and the pellet was air dried before being re-suspended in enough Tris-EDTA (TE) buffer (pH 8.0) to give a 100 µM solution of thiol-modified ssDNA.

### 2.4. Pre-Treatment of the Gold Working Electrode

The working electrode used for electrochemical measurements was a gold coated glass substrates. The gold electrode was annealed to achieve the structural reconstruction of the gold surface into the Au(111) configuration for immobilization purposes. The gold electrode was passed through a flame until the corners glowed red, then allowed to cool. This was repeated 2–3 times. The annealed gold electrode was allowed to cool for at least 30 min before the thiol-ssDNA capture probe was incubated on the surface.

### 2.5. Thiol-ssDNA Probe Immobilization on the Gold Electrode Surface

The ssDNA capture probe (200 µL, 1 µM) was pipetted on to the surface of the gold working electrode and incubated for 90 min at 37 °C. The remaining capture probe solution was then decanted from the surface and the gold electrode was washed with nuclease free ultra-pure water and air dried. Once dried, adsorptive stripping voltammetry (AdSV) was used to confirm the presence of the gold–thiol bond. The dried working electrode was transferred to an electrochemical cell along with a platinum (Pt) counter electrode and an Ag/AgCl reference electrode. NaOH (250 mL, 0.1 M) was made up in distilled water and degassed with nitrogen for at least 30 min. Once degassed, NaOH (15 mL, 0.1 M) was added to the electrochemical cell. All three electrodes in the cell were connected to a Metrohm autolab potentiostat, PGSTAT 30 (Eco Chemie, Holland) and the stripping analysis was performed using the Metrohm autolab Nova 2.1 software. The stripping voltammetry parameters were two consecutive scans with a scan rate of 50 mV/s and a potential scanning range of 0 to −1.2 V.

### 2.6. Electrochemical Confirmation of DNA Hybridization

For the DNA hybridization study, cyclic voltammetry (CV) and EIS were employed. Ferri/ferrocyanide (5 mM) in 100 mM KCl was utilized as a redox probe in each electrochemical study. The redox probe was degassed with nitrogen for at least half an hour before being used in each measurement. The electrochemical cell was set up with the modified gold working electrode, a Pt counter electrode and an Ag/AgCl reference electrode. Once set up, the degassed redox probe was added into the cell and scans were recorded. For EIS, a 0.3 V potential was applied and frequencies of 10,000 Hz to 0.1 Hz were applied with a frequency step of 10 points per decade. All impedance spectra were obtained using a frequency response analyzer (FRA). The parameters for the CV scans were as follows; number of scans: 2, scan rate: 50 mV/s, with a potential range 0.8 to −0.4 V.

## 3. Results

### 3.1. Target Identification and Capture Probe Design

The ssDNA capture probes were designed from 16 s ribosomal ribonucleic acid (rRNA) sequences because there have been multiple nucleotide-based sensors that have successfully used probes based on these sequences [37,38,39]. This is because rRNA sequences have been found to contain less variation and the specificity of the probe can be more readily adjusted than probes based on whole DNA sequences [40,41].

The chosen target sequences were modified to contain up to four mismatched nucleotides to increase selectivity. The ClustalW alignment tool showed that the modified sequences only aligned with their bacterial group after the modification. Additionally, the modified target sequences were checked for alignment with *Lactobacillus* spp. which is a common bacterial species that inhabit the horse’s gut. The search showed that none of the modified sequences matched up with *Lactobacillus* spp. meaning that there will be less chance of the sensor giving a false reading. The original target sequences, the modified target sequences, and the complementary capture probes are presented in Table 1.

### 3.2. Gel Electrophoresis Study of DNA Hybridization

Before testing the hybridization reactions between capture probe and target sequence combinations on gold, they were tested in solution. This was to make sure there was an interaction between the ssDNA without the added complication of binding the capture probe to gold first. Two types of capture probe were initially tested for hybridization with the target sequences in solution: one being capture probes that had been modified with a thiol group and others that were unmodified forms of the capture probes. This was done to test whether hybridization would firstly occur with the unmodified capture probe and then, if successful, further tests with the modified form would be performed.

### 3.3. DNA Hybridization Study with the Unmodified Capture Probes

**Figure 2:**
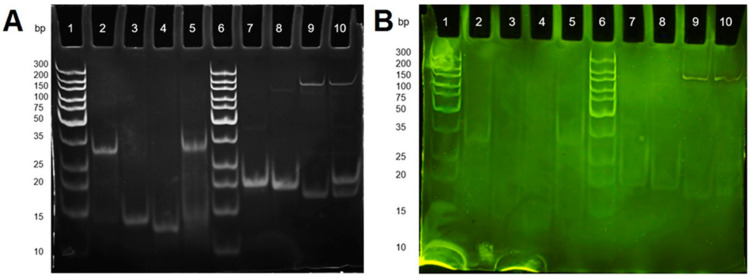
(**A**) Polyacrylamide gel electrophoresis of hybridisationhybridization between the two unmodified capture probe and target sequence combinations stained using ethidium bromide: SECP1 (unmodified) with *Streptococcus equinus* target 1 (SET1) and *Mitsuokella jalaludinii* capture probe 1 (MJCP1) (unmodified) with *Mitsuokella jalaludinii* target 1 (MJT1); and (**B**) Polyacrylamide gel electrophoresis of hybridization between the two unmodified capture probe and target sequence combinations stained with acridine orange: *Streptococcus equinus* capture probe 1 (SECP1) (unmodified) with SET1 and MJCP1 (unmodified) with MJT1: where Lanes 1 and 6 are Gene Ruler ultra-low range DNA ladders; Lanes 2 to 5 are: SECP1 (modified), SECP1 (unmodified), SET1, and a hybridization reaction of SECP1 (unmodified) with SET1, respectively; and Lanes 7 to 10 are: MJCP1 (modified), MJCP1 (unmodified), MJT1, and a hybridization reaction of MJCP1 (unmodified) with MJT1, respectively.

Two out of four of the capture probe and target combinations were tested in unmodified forms, one from the SE bacteria and one from the MJ bacteria. Both hybridization pairs were monitored by polyacrylamide gel electrophoresis (Figure 2A). SE capture probe 1 (SECP1) was paired with its corresponding target SE target 1 (SET1) and MJ capture probe 1 (MJCP1) was paired with its corresponding target MJ target 1 (MJT1) for the hybridization study with unmodified capture probes.

Each hybridization reaction had corresponding controls on the gel with lanes containing exclusively capture probe and exclusively target. This was primarily to compare with the hybridization reaction, but it also allowed comparison with the DNA ladder to see if the capture probe and target were of the correct size. The ladder used here contained dsDNA and so the comparison with the ssDNA was not accurate. However, the main purpose of the gel was to see if there was a difference in brightness and position in the gel that would arise from the hybridization reaction.

Theoretically, the binding of the capture probe and target would give rise to a brighter band that would sit higher in the gel. The increased brightness would occur because ethidium bromide (EtBr) binds more effectively to dsDNA due to the way it intercalates between the hydrogen bonded strands of the DNA and binds to both strands [42,43]. From this way of binding, it is less likely that the EtBr stain would bind effectively to ssDNA because it would need to fold over on itself for this type of binding to occur. As the ssDNA may not always be in the conformation it needs for EtBr staining, this could be the reason why there is less fluorescence when visualized under UV light. Additionally, the position of dsDNA should be higher in the gel than ssDNA because the size and flexibility of the ssDNA allow for movement through the pores of the gel more easily, and therefore, they will move further down the gel in the allotted time, in comparison to dsDNA [44]. Having rigidly bound strands and being of larger size, the dsDNA should find it a lot more difficult to move through the pores of the gel. For the SE1 side of the gel, these theories appeared to be confirmed due to the increased brightness of the hybridized band and the lower distance it was able to travel through the gel causing the band to appear higher in the gel when compared to the SECP1 (unmodified) and SET1 controls (lanes 3 and 4, respectively). There is also a small shadow of a band lower down the SE1 hybridization lane which appears to be some of the SECP1 and SET1 that have not hybridized together. The decreased intensity and sharpness of the band, compared to the band higher in the gel, shows that there is less DNA present and so most of the ssDNA has bound together and is present in the higher band.

On the MJ1 side of the gel, the result differed. The hybridization in lane 10 appears to be unsuccessful because the capture probe and target have separated in similar places compared to the MJCP1 and MJT1 control bands. This indicates there has been little to no hybridization between the capture probe and target. Additionally, there was a band present in both lanes 9 and 10 at approximately 150–200 bp. As this band is present in both the MJT1 control and the MJCP1 (unmodified)/MJT1 hybridization lane, it must have been a feature of the target sequence and most likely due to strands of the target DNA binding together. Further studies reinforced this theory because the hybridization reaction between MJCP1 (unmodified) and MJT1 was repeated and heated to allow the bonds between the strands of the target to denature. The reaction mixture was then cooled to allow hybridization between the capture probe and target. Results showed that the band at 150–200 bp was not present after this process but the hybridization still appeared unsuccessful.

Lanes 2 and 7 contain the modified version of SECP1 and MJCP1 and were added to the gel to confirm hybridization because they were not used for any hybridization studies in this gel. Their purpose was to see if there were any differences between the positions of the unmodified and modified capture probe bands in the gel. Both of the modified capture probes moved through the gel less effectively than the unmodified version. This is most likely due to the thiol modification interacting with the pores of the gel, increasing the drag when being pulled through the pores under the influence of the applied voltage, resulting in the modified capture probe moving at a slower rate through the gel.

Another gel was run containing identical samples but with different staining (Figure 2B). Instead of the ethidium bromide stain, acridine orange (AO) was utilized due to its differential staining ability. Acridine orange is a metachromatic dye because of its ability to considerably change the wavelength it absorbs at in the visible spectrum when staining [45]. Ethidium bromide is a very commonly used stain for DNA separation but it is unable to effectively differentiate between single stranded and double stranded DNA apart from the few assumptions that can be made based on the indications of a brighter band or DNA that has more limited movement through the gel as presented previously [46]. Contrastingly, AO can stain single and double stranded nucleic acids differently. It has been suggested that AO intercalates in between adjacent base pairs at a position that is perpendicular to the axis of the double helical nucleic acid structure [47,48]. When this binding event occurs in dsDNA, the result is a green fluorescence at 530 nm. When AO interacts with single stranded nucleic acids, the result is an electrostatic binding between the AO and the phosphate back bone present in the nucleic acid which gives rise to red fluorescence at 640 nm. In Figure 2B, the idea was to exploit AO for its differential fluorescence depending on whether it is binding to single or double stranded DNA. This would have given more information than the previous gel on whether the hybridization was occurring because there would be a more visual clarification. Additionally, AO would give information on the nature of the capture probe and target sequences.

However, the desired effect of staining the dsDNA green and the ssDNA red was unsuccessful as all of the stained bands apart from two were green. The two bands at the top of lanes 9 and 10, which were previously theorized as being target strands bound to each other in solution, were observed as red when observed by the eye although the camera was unable to pick this up. This reinforces the idea stated previously about the target strands binding together but it also shows that the strands are not bound in the same way as two ssDNA strands are in dsDNA. Therefore, the target strands must be binding together in a different way that still allows the dye to bind in the way it does to ssDNA. It appears that the lower base pair ssDNA strands were unable to interact with the dye correctly because the bands are barely visible on this gel and this could be connected to the small size of the ssDNA strands. Additionally, these ssDNA strands could be in a hairpin conformation, meaning some bases in an individual strand may be bound together allowing the AO to bind in the same way as it would to dsDNA.

### 3.4. DNA Hybridisation Study with Thiol-Modified Capture Probes

**Figure 3:**
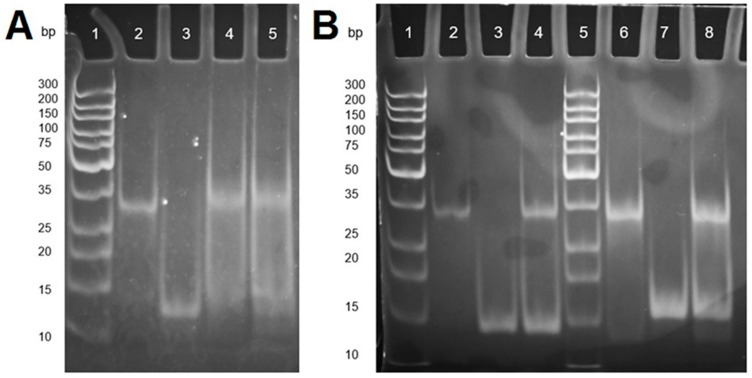
(**A**) Polyacrylamide gel electrophoresis of the hybridization of SECP1 (thiol-modified) with SET1: Lanes 1–5 in respective order are: GeneRuler ultra low range DNA ladder, SECP1 (modified), SET1, hybridization of SECP1 (modified) with SET1 (at RT), and the same hybridization reaction carried out on ice; and (**B**) Polyacrylamide gel electrophoresis of hybridization reactions for SECP2 (mod) with SET2 and MJCP2 (mod) with MJT2: Lanes 1 and 5 contain the GeneRuler ultra low range DNA ladder; and Lanes 2 to 4 and lanes 6 to 8 contain: SECP2 (mod), SET2, SE2 hybridization reaction, MJCP2 (mod), MJT2, and the MJ2 hybridization reaction, respectively.

After the indication of successful hybridization between SECP1 (unmodified) and SET1 from the previous gel, the next step was to test the thiol-modified version of SECP1 with SET1 in the same way. The purpose of the thiol modification was to be able to exploit the affinity between Sulphur and gold to create a self-assembled monolayer (SAM) of capture probe on a gold surface [26]. For this reason, it was clear that the thiol-modified version of the capture probe must be tested for hybridization in solution before attempting it on the gold surface. The result was again monitored by polyacrylamide gel electrophoresis (Figure 3A).

Figure 3A indicates that the hybridization reaction had been successful. The reaction didn’t show two distinct bands but instead was mostly one band that was brighter and at a higher position in the gel when compared to the controls. Therefore, as discussed previously, the gel indicated successful hybridization with the inclusion of a thiol modification on the capture probe. This gel also gave information on the conditions in which hybridization would occur. The reactions which were carried out at room temperature (approximately 22 °C) and on ice (approximately 0–1 °C) gave very similar results, meaning that either condition could be used for further experiments.

Hybridization studies for the other two capture probe and target combinations were also explored on gels; SECP2 (mod) with SET2 and MJCP2 (mod) with MJT2 (Figure 3B). In the two hybridization reactions, two bands were visualized which were evidence of unsuccessful binding concerning the previous experiments and theories. Here, both of the capture probes and targets had been separated into their respective bands, similar to how MJCP1 and MJT1 were separated in previous experiments. Having a high concentration of hairpin conformation ssDNA could be a possible reason for the failure of the strands to hybridize together because the bases are less exposed to each other resulting in a lower chance of binding. As a result of the polyacrylamide electrophoresis studies, only one of the four capture probe and target combinations were taken forward for further study: SECP1 and SET1. This combination showed binding to the target with both the unmodified and thiol-modified forms in solution, whereas the other three combinations showed no indication of hybridization.

### 3.5. Stripping Voltammetry Study of ssDNA-SH-Gold Bond

**Figure 4:**
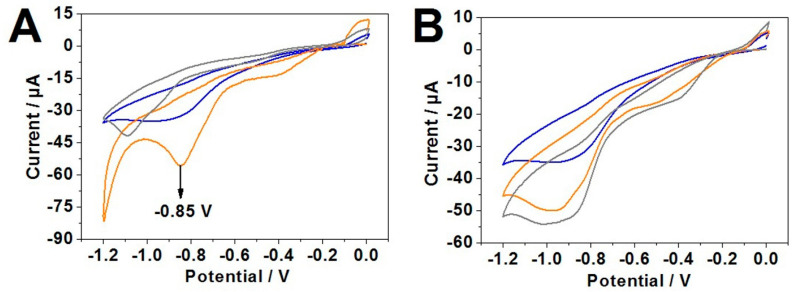
(**A**) Stripping voltammogram of SECP1 (modified) and SET1 on a gold working electrode. Blank gold working electrode (blue curve), SECP1 (modified) incubated gold working electrode (orange curve), and SECP1 (modified) incubated gold working electrode with the addition of SET1 (grey curve); and (**B**) Stripping voltammetry of blank gold working electrode (blue curve), gold working electrode incubated with the unmodified SECP1 (orange curve), and gold working electrode incubated with the unmodified SECP1 and then SET1 (grey curve).

The next step in this work was to obtain evidence of the thiol-modified SECP1 binding to a gold surface. For this, the capture probe was incubated on a gold electrode and then it was electrochemically stripped from the surface, via the reduction of the thiol–gold bond generated in a SAM. The AdSV was employed to study the adsorption/desorption of the capture probe layer on the surface of the working electrode in the presence of NaOH due to its basicity. Three scans were taken: one of the blank gold working electrodes, one of the gold working electrodes with exclusively capture probe on the surface, and another that contained the target as well as the capture probe (Figure 4A).

The expected potential for the thiol desorption peak is around −0.97 V as reported elsewhere [xvi]. From the voltammogram, it was evident that there was no thiol reduction peak on the blank gold working electrode (Figure 4A, blue curve). Contrastingly, when there is an addition of the capture probe, there is a clear desorption peak at around −0.85 V (Figure 4A, orange curve), which is approximately 0.12 V less negative than in the literature [xvi]. This is most likely due to the amount of time the capture probe has incubated on the surface of the gold. In the literature, the moiety containing the thiol was incubated on the gold surface for 48 h at 4 °C, whereas in this work the capture probe was incubated for 90 min at 37 °C. The bonds formed between the thiol and the gold surface at a lower temperature and over a longer period may be more strongly adsorbed to the gold surface, therefore increasing the degree of negativity of voltage required for the stripping to take place. This could occur due to the adsorption of the thiol onto the gold being more kinetically favorable at lower temperature and over a longer period of time because the thiol containing compounds are more able to move around and order themselves more efficiently on the gold surface.

The thiol desorption peak was a clear indication that the thiol modification was interacting with the gold surface and therefore the capture probe was bound to the surface. If some of the ssDNA was bound to the gold surface differently, i.e., if the bases were electrostatically bound to the surface, the strands of ssDNA may be stripped under less of a voltage due to the weaker interactions and at varying voltages. An additional scan was performed where the capture probe-modified gold surface was incubated with the target to see if there were any differences from the previous scans (Figure 4A, grey curve). This scan showed that the characteristic thiol desorption peak was not present. Instead, there is a small desorption peak at around −1.1 V. which may correspond to the desorption of the thiol bound hybridized dsDNA. This desorption peak is approximately 0.3 V more negative than the thiol desorption peak of the capture probe, exclusively. This could indicate that the capture probe and target could have bound together on the gold surface and the resulting dsDNA may be more difficult to strip from the surface, requiring a more negative potential for the thiol desorption to take place.

Additional to the previous stripping data, another set of scans were taken with the unmodified SECP1 and with SET1 to compare and contrast the results with unmodified SECP1 (Figure 4B). This stripping data contrasts with the previous set of scans, which was expected due to SECP1 lacking a thiol modification. There is no thiol and therefore no thiol desorption peak present; however, there are still strands of SECP1 and SET1 binding, electrostatically, to the surface in a non-specific way. This is shown in the scans by the trend of increasing negative current going from the blank scan toward the target scan. The current is more negative in the SECP1 scan (Figure 4B, orange curve) than the blank gold working electrode (Figure 4B, blue curve) because there are some loosely bound capture probe strands on the surface. When met with a sufficiently negative potential for cathodic transfer to take place, they are stripped off the surface. This is the reason for the increase in current here. Furthermore, when the target is added to a capture probe-modified gold electrode (Figure 4B, grey curve), the stripping yields a slightly larger negative current because in this case there are loosely bound SECP1 as well as some loosely bound SET1 where some may have hybridized and some may be separated. With the addition of the target, there are more strands of DNA on the surface of the gold electrode and therefore a more negative current is generated when the stripping occurs at the appropriately negative potentials.

It can also be seen that the stripping peaks in Figure 4B are much broader than the thiol desorption peaks in Figure 4A. This could be because when the ssDNA capture probe is bound mainly by thiol, the desorption will be more uniform. The capture probes will all be adsorbed to the gold surface in a very similar way so most will desorb at a very similar potential, resulting in a narrow peak with a large current. Contrastingly, when there is no thiol present and the capture probe binding to the surface is by electrostatic interactions, the binding is far more inconsistent because all of the strands will be binding by different amounts and in different arrangements in space. This results in a broader scan because the inconsistently bound ssDNA strands are being stripped at varying potentials which results in a broader stripping potential range and a broader current peak.

### 3.6. Cyclic Voltammetry Study of DNA Hybridization

**Figure 5:**
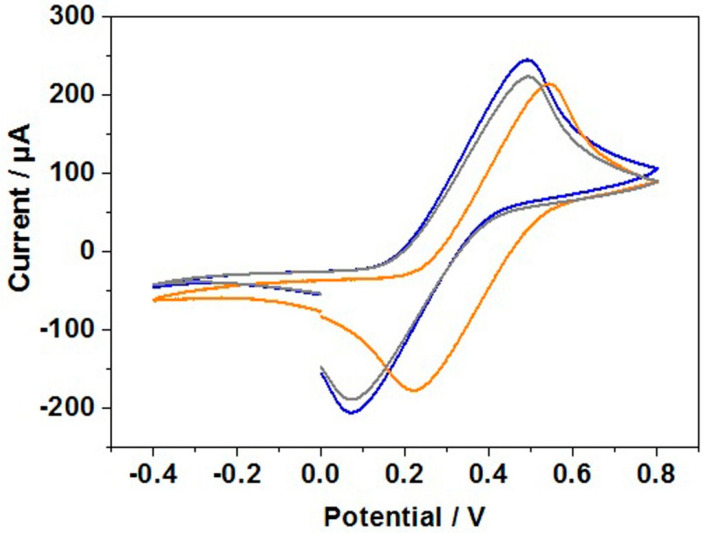
Cyclic voltammetry scans in 5 mM potassium ferri/ferrocyanide of blank electrode gold (blue curve), gold electrode incubated with SECP1 (mod) (orange curve), and gold electrode incubated with SECP1 (mod) and SET1 (grey curve).

Following the information gathered from the studies using AdSV, CV was performed to confirm hybridization of the capture probe and target on the gold working electrode. The redox probe [Fe(CN)_6_]^3−/4−^ was used for the CV scans. Scans of blank gold electrode, gold electrode incubated with capture probe, and gold electrode incubated with capture probe and target, were taken as shown in Figure 5.

The reduction and oxidation peaks are at their highest points during the scan of the blank gold working electrode (Figure 5, blue curve). This suggests the flow of electrons between the redox probe and the gold is larger when the surface of the gold is not modified by any surface adsorbed material. This is because there is a larger surface area of the gold electrode available for redox activity. With the addition of the capture probes, as shown in Figure 5 (orange curve), the oxidation and reduction peaks are lower than that of the blank gold electrode scan which shows a lower redox activity when the gold electrode surface is modified with the capture probe.

Due to the flexible nature of the ssDNA, the capture probe can bind through its thiol modification but also non-specifically because the ssDNA can lay across the gold electrode in various arrangements. The resultant tight packing of ssDNA on the gold surface hinders the pathway of the redox probe to the gold surface which in turn reduces the redox activity. However, when the target is added, the reduction/oxidation peaks are higher (Figure 5, grey curve) than that of the capture probe scan although not as high as the current seen in the blank gold scan. An explanation for this could be that when the target is added, the capture probe has hybridized with the target to form more rigid hydrogen bonds to form the duplex DNA. When this hybridization occurs, the amount of ssDNA non-specific binding is reduced because the ssDNA can effectively lift off the gold electrode surface so the bases can preferentially bind with the target leaving only the thiol bond to the gold in place [49,50,51]. This frees up space on the gold surface meaning that the redox probe can reach the gold electrode and undergo redox reactions more effectively than before the addition of the target. This increases the reduction and oxidation peaks when compared with the capture probe only scan. However, there are still parts of the gold surface that are occupied with a surface adsorbed species (dsDNA) and so the redox activity and therefore the oxidation/reduction peaks are still lower than the blank gold scan. This behavior has also been reported in recent literature [49]. However, the superiority of the fabricated DNA biosensor over reported work elsewhere [49] includes: (a) four capture probe/target combinations being tested, (b) polyacrylamide gel electrophoresis being used for hybridization screening, and (c) stripping voltammetry being employed to demonstrate that thiol modified ssDNA consistently bind over gold working electrode.

### 3.7. EIS Confirmation of DNA Hybridization

**Figure 6:**
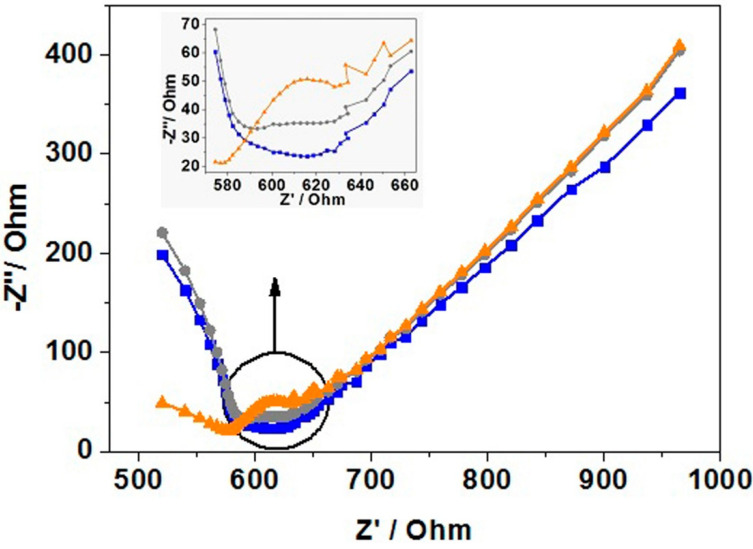
The initial Nyquist plot obtained from the electrochemical impedance spectroscopy (EIS) studies. Blank gold electrode (blue curve), gold electrode incubated with SECP1 (mod) (orange curve), and gold electrode incubated with SECP1 (mod) with SET1 (grey curve).

The indications of hybridization demonstrated previously were followed by EIS studies to see if the hybridization could be confirmed. The EIS studies were performed with the same redox probe and electrochemical cell setup as the CV. Additionally, the incubation technique and scan content were consistent with the CV scans. Three scans were taken, one of the blank gold electrodes, one of a gold electrode was incubated with SECP1 (modified), and a third gold electrode that was incubated with SECP1 (modified) and afterwards SET1 (Figure 6).

The charge transfer resistance at the surface of the working electrode is inferred by the diameter of the semicircle present in the Nyquist plot [16]. From the scans, it was clear that the lack of a semicircle present in the scan of the blank gold electrode showed low amounts of charge transfer resistance (Figure 6, blue curve). When incubated with SECP1, the charge transfer resistance increased substantially (Figure 6, orange curve), then decreased with the addition of the target (Figure 6, grey curve). The trend in these scans was expected when compared to the literature because they showed similar behavior [48]. Additionally, these EIS scans reinforce the CV data presented previously. The charge transfer resistance of the blank gold electrode was expected to be low because the surface had not been exposed to any surface-active species and therefore the redox probe was able to interact with the gold surface without any major hindrances. As previously discussed, the ssDNA capture probe’s ability to fold and move more freely than dsDNA means there is more chance of the ssDNA blocking the surface by lying across it in various conformations. This gives a large impedance because the charge transfer between the redox probe and the gold working electrode is hindered due to the ssDNA blocking the probe’s path to the electrode. When the target is added and hybridization takes place, the ssDNA capture probe on the surface is lifted to bind to the target and the impedance is lowered because more of the gold surface is exposed to the redox probe and more charge transfer can occur [47,49,50]. This lower charge transfer resistance was seen in the scan that had both the capture probe and target (grey) on the surface by the smaller diameter of the semicircle.

## 4. Conclusions

This study demonstrated that only one out of the four initial target and capture probe combinations, SECP1/SET1, were able to hybridize successfully. This is shown by polyacrylamide gel electrophoresis gels where each of the combinations were tested for the hybridization. Both the thiol-modified and unmodified versions of the SECP1 capture probe were able to successfully hybridize with the corresponding SET1. When the SECP1 and SET1 studies were moved onto the gold working electrode, it was evident from AdSV that the unmodified was not bound in the same way as the thiol-modified as there was no sharp desorption peak present, whereas there was for the modified capture probes due to its thiol modification binding to gold. Further CV and EIS measurements supported the binding of thiol modified SECP1 over the electrode surface and hybridization with SET1. These results show that the proposed electrochemical biosensor could be used for equine hindgut acidosis monitoring and detection to help prevent laminitis. Linear range and limit of detection need to be evaluated, and for robust application, the proposed biosensor should be tested with real samples for the detection of the non-synthetic bacterial DNA target.

## Figures and Tables

**Table 1:** Four single stranded deoxyribonucleic acid (ssDNA) target sequences, two from the *Streptococcus equinus* (SE) bacteria and two from the *Mitsuokella jalaludinii* (MJ) bacteria, identified and modified to contain mismatched base pairs to increase binding selectivity. Four ssDNA capture probe sequences designed complementary to the modified target sequences.

Oligonucleotide	Position	Size (bp)	Sequence
SE target sequence (1)	816–836	21	GGG TCC TTT CCG GGA CTC AGT
SE mod target sequence (1)		21	GGG ACC TTT CCG GGC TTC AGT
SE capture probe (1)		21	CCC TGG AAA GGC CCG AAG TCA
SE target sequence (2)	642–662	21	AAG GGG AGA GTG GAA TTC CAT
SE mod target sequence (2)		21	AAG GGT AGA CCG GAA TTC CAT
SE capture probe (2)		21	TTC CCA TCA GGC CTT AAG GTA
MJ target sequence (1)	650–675	25	CCG TGA GGG GAT GGA AAC TAT CTT T
MJ mod target sequence (1)		25	CCG TGA GGG GAT AAA CAC TAT CTT T
MJ capture probe (1)		25	GGC ACT CCC CTA TTT GTG ATA GAA A
MJ target sequence (2)	1180–1200	25	TCC TTT GTT GCC AGC ACG CAA TGG T
MJ mod target sequence (2)		25	TCC TTT GTG CCC AGC GCG CCA TGG T
MJ capture probe (2)		25	AGG AAA CAC GGG TCG CGC GGT ACC A

## Data Availability

Not applicable.
